# Neurophysiological and biosignal data for investigating occupational mental fatigue: MEFAR dataset

**DOI:** 10.1016/j.dib.2023.109896

**Published:** 2023-12-09

**Authors:** Seyma Derdiyok, Fatma Patlar Akbulut, Cagatay Catal

**Affiliations:** a Department of Computer Engineering, Yıldız Technical University, Istanbul, Turkey; b Department of Software Engineering, Istanbul Kültür University, Istanbul, Turkey; c Department of Computer Science and Engineering, Qatar University, Doha, Qatar

**Keywords:** Physiological signals, Mental workload, Human mental performance, Cognitive fatigue

## Abstract

The prevalence of mental fatigue is a noteworthy phenomenon that can affect individuals across diverse professions and working routines. This paper provides a comprehensive dataset of physiological signals obtained from 23 participants during their professional work and questionnaires to analyze mental fatigue. The questionnaires included demographic information and Chalder Fatigue Scale scores indicating mental and physical fatigue. Both physiological signal measurements and the Chalder Fatigue Scale were performed in two sessions, morning and evening. The present dataset encompasses diverse physiological signals, including electroencephalogram (EEG), blood volume pulse (BVP), electrodermal activity (EDA), heart rate (HR), skin temperature (TEMP), and 3-axis accelerometer (ACC) data. The NeuroSky MindWave EEG device was used for brain signals, and the Empatica E4 smart wristband was used for other signals. Measurements were carried out on individuals from four different occupational groups, such as academicians, technicians, computer engineers, and kitchen workers. The provision of comprehensive metadata supplements the dataset, thereby promoting inquiries about the neurophysiological concomitants of mental fatigue, autonomic activity patterns, and the repercussions of a cognitive burden on human proficiency in actual workplace settings. The accessibility of the aforementioned dataset serves to facilitate progress in the field of mental fatigue research while also laying the groundwork for the creation of customized fatigue evaluation techniques and interventions in diverse professional domains.

Specifications Table

**Table:** 

Subject	Data Science Computer Science Health and medical sciences
Specific subject area	Mental fatigue analysis, Mental fatigue recognition, Signal Processing, Biosignal classification
Data format	Raw, Analysed
Type of data	Table
Data collection	The physiological data utilized in this dataset was obtained through the utilization of two distinct devices and software applications. The NeuroExperimenter app was used to get the NeuroSky MindWave brain signals, while the E4 Realtime app was used to get the Empatica E4 biosignal data. During the EEG data collection process, the study participants were equipped with the NeuroSky MindWave headset, and the NeuroExperimenter application was utilized to effectively capture and record the EEG signals. The application facilitated the contemporaneous recording of electroencephalographic data by employing a single-channel electrode affixed to the cranial region. The EEG signals were recorded with a sampling rate of 1 Hz. During data collection, the Empatica E4 wristband was utilized to capture physiological signals including BVP, EDA, HR, skin temperature, and a 3-axis accelerometer. The Empatica-provided "Realtime" application was employed to capture the physiological signals stemming from the E4 device. The subjects donned an E4 wristband on their non-dominant wrist, which facilitated the uninterrupted recording of their physiological indicators throughout their professional engagements. The BVP was recorded using a sampling frequency of 64 Hz to effectively capture any variations in the signal. EDA was recorded by sampling the signal at a frequency of 4 Hz. Human HR and body temperature were obtained as instantaneous measurements at regularly spaced intervals, while the accelerometer was represented by the ACC signal and sampled at a frequency of 32 Hz to capture and analyze movement and activity levels. Thorough configuration and adjustment procedures were executed on every apparatus and software program to guarantee the precise acquisition of data. Physiological signals recorded in separate files were combined according to different sample rates using resampling techniques, and sub-datasets of three different sizes were created. The questionnaire data includes demographic information such as age, occupation, education, employment status, etc. of the participants and mental fatigue scores calculated according to their responses to the Chalder Fatigue Scale. In the Chalder Fatigue Scale, the responses of the participants were made according to a 4-point Likert scale in the range of 0–3. The participant's responses to the questions on the scale were scored as 0 if less than usual, 1 if as usual, 2 if more than usual, and 3 if much more than usual.
Data source location	Institution: Istanbul Kultur University, Department of Computer Engineering City/Town/Region: Istanbul, Bakırköy, Ataköy Country: Turkey Latitude and longitude (and GPS coordinates, if possible) for collected samples/data: 41° 5 7.3284′’ N 29° 2 22.3836 ’ E
Data accessibility	Repository name: Mendeley Data Data identification number: 10.17632/z3g26tphnv.5 Direct URL to data: https://data.mendeley.com/datasets/z3g26tphnv/5

## 1 Value of the Data

• This dataset presents advantageous insights concerning the physiological signals implicated in mental fatigue. This offers a heightened comprehension of the fundamental mechanisms and dynamics of fatigue in diverse occupational environments.
• Scholars within the domains of psychology, neuroscience, and human factors stand to gain significant benefits from the utilization of such data in probing the interplay between physiological responses and mental fatigue, thereby advancing the development and implementation of effective tools and interventions for assessing and addressing fatigue.
• The dataset possesses the potential for examining the effects of varying occupations on mental exhaustion, ultimately enabling precise interventions and workplace arrangement tactics to enhance output and welfare.
• The presented dataset holds the potential to serve as a standardized measure for the purpose of developing machine learning algorithms or predictive models that enable real-time detection of mental fatigue. This, in turn, promises to strengthen workplace safety and enhance performance management.
• Scholars have the opportunity to employ these data for the purpose of investigating the relationship between mental fatigue and additional variables, including cognitive function, level of stress, or outcomes linked to employment, thus resulting in a thorough comprehension of the implications of fatigue in a variety of contexts.

## 2 Data Description

Mental fatigue, a pervasive phenomenon in various domains of our existence, is characterized as a form of objective and subjective exhaustion that arises from prolonged engagement in cognitive activities [1]. It has implications [2], [3], [4], [5], [6] on emotions, behavior, physical well-being, and social interactions. The effects encompass a spectrum of emotions, including inexplicable rage and melancholy, as well as a reluctance to engage in social settings. Analyzing and recognizing this particular sort of fatigue, which is an emotion that diminishes situational awareness, the fundamental aspect of human and occupational safety [7], might help minimize any negative consequences. Currently, the focus of research on emotion recognition often revolves around analyzing physiological information [8,9] utilizing artificial intelligence [10]. These experiments demonstrated a significant success rate and found that signals originating from locations directly influenced by the parasympathetic nervous system were more favored. Hence, this dataset was generated with the purpose of examining mental exhaustion through the analysis of physiological signals. This study offers a chance to meticulously investigate the impact of the neurological system, which is extremely responsive to external stimuli, on mental exhaustion. By examining the different signals from the brain, heart, and skin, it is possible to examine them individually and deduce their correlation with mental tiredness. The subjects were assessed for mental fatigue using a fatigue scale. The Chalder Fatigue Scale [11,12] was employed in this phase, serving as the definitive measure in the study. The study conducted a test to assess the validity and reliability of the scale, and it yielded satisfactory results [13]. The questions on this scale serve to assess both mental and physical weariness. Fatigue states were assessed using a 4-point Likert scale. The participants’ mental weariness was categorized based on these scores. One additional advantageous aspect of this study is the provision of annotated data. The MEFAR dataset is organized in two main components.

**Fig. 1:**
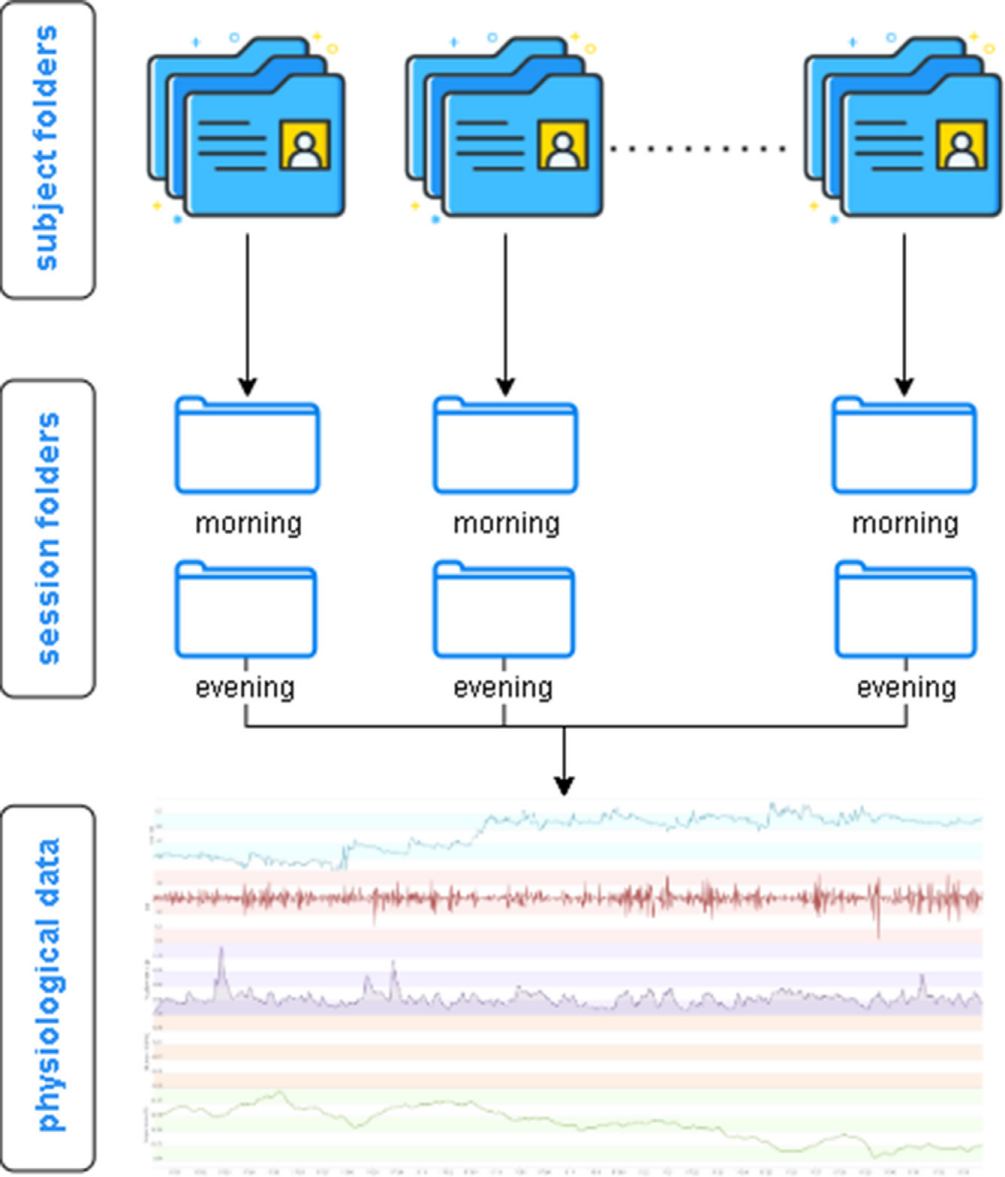
The folder structure is visually represented through the use of illustrations, depicting the organization of raw data.

• The first component is the folder holding the raw signals of all subjects, called MEFAR_raw. This keeps all the raw signals of each participant in a folder created according to the identification number assigned to each participant as depicted in Fig. 1. Each participant folder contains morning and evening subfolders that store physiological signal data such as EEG, BVP, EDA, TEMP, HR, ACC. In addition to this file, there is an excel file named general_info containing demographic information and Chalder Fatigue Scale scores of the participants as illustrated in Table 1*.* Also, this excel file contains subjects ‘responses to the Chalder Fatigue Scale (CFS) both morning and evening sessions in separate sheets. The responses are summed according to a 4-point Likert scale. These answers and their points are as follows: less than usual 0 point, no more than usual 1 point, more than usual 2points and much more than usual 3 points. The cutoff value was set as 12 and scores above this value in the excel were colored in red and scores below this value in green.

**Table 1:** Description of survey data.

Data	Data type	Data description
Subjects	String	Subject id number
Age	Integer	Subject's age
Gender	String	Subject's gender
Education	String	Subject's last education that received
Employment	String	Subject's working type (full or part time)
Marital Status	String	Subject's marriage status
Smoking	Boolean	Whether subject is smoking
Sleep Status	Boolean	Whether subject has regular sleep
Salary	Integer	Subject's salary per month in Turkish lira
Chronic Disease	Boolean	Whether subject has chronic disease
Drug Status	Boolean	Whether participants take medication regularly or not
Gym Status	Boolean	Whether subject has regular gym
Duration of Employment	Integer	indicates the participants’ working hours per day
Last Sleep Time	Integer	indicates how many hours the participant slept the night before participating in the experiments
Morning MF State	Integer	Indicates subject's mental fatigue measured in the morning
Evening MF State	Integer	Indicates subject's mental fatigue measured in the evening

• The second component is MEFAR_preprocessed folder that keeps normalized, upsampled and downsampled physiological data. The signals obtained have different frequencies. The signals must be adjusted to a common sample rate. In this direction, 3 different sampling techniques were applied according to the frequency values of 1 Hz, 32 Hz and 64 Hz. We completed the preprocessed dataset folder by naming the dataset that we sampled in accordance with the frequency of 1 Hz as MEFAR_DOWN, the dataset that we sampled in accordance with the frequency of 32 Hz as MEFAR_MID and finally the dataset that we sampled in accordance with the frequency of 64 Hz as MEFAR_UP. Each MEFAR file contains BVP, EDA, TEMP, ACC, HR, Delta, Theta, Alpha1, Alpha2, Beta1, Beta2, Gamma1, Gamma2, Attention, Meditation and class values. Class column is the label of the observation and 1 means mental fatigue and 0 means not mental fatigue.
• The MEFAR_DOWN.csv file, which contains the data sampled according to 1HZ, contains a total of 27,570 data. Of these, 14,464 are labelled as mental fatigue and 13,106 as not mental fatigue.
• The MEFAR_MID.csv file, which contains the data sampled according to 32 Hz, contains 923,298 data in total. Half of these data are labelled as mental fatigue and the other half as not mental fatigue.
• The MEFAR_UP.csv file, which contains the data sampled according to 64 Hz, contains 1,846,590 data in total. Half of these data are labelled as mental fatigue and the other half as not mental fatigue.

## 3 Experimental Design, Materials and Methods

1. **Research design**

**Fig. 2:**
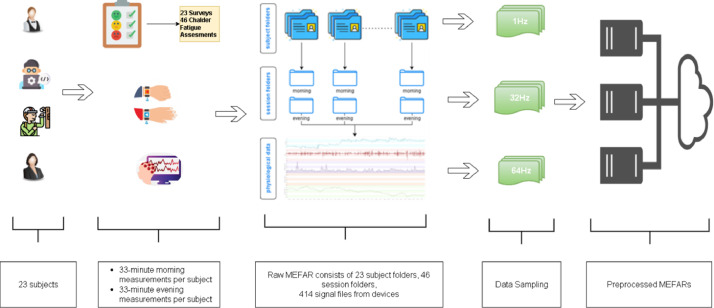
Research design of acquiring data.

The first task to create the MEFAR dataset, which was created to analyze mental fatigue based on physiological signals, was to determine the groups of people to collect data, as shown in Fig. 2. In our research, we paid attention to the fact that the participants whose signals were collected were active employees. For this reason, full-time employees between the ages of minimum 18 and maximum 50 were selected as subjects. After the individuals were identified, physiological signal measurements were taken from the participants in two sessions, one in the morning and one in the evening. Measurements in the morning sessions were taken between 8 am and 12 noon, while evening measurements were taken between 4 pm and 6 pm. The duration of each measurement was planned to be at least 33 and at most 36 min. Considering the possibility of being less fatigued in the morning and more fatigued in the evening, the data collection process was carried out in the morning and evening. Thus, it is aimed to label the signal data in a balanced way. In the first measurement, the participants were asked to answer the demographic information questionnaire and the questions on the Chalder Fatigue Scale. In the second measurement, participants were expected to answer only the fatigue scale. During the measurement, the participants were reminded to continue with their routine work patterns. The aforementioned signals in the dataset were received from two different wearable devices. These are the Empatica E4 smart wristband worn on the participants' left wrist and NeuroSky Mindwave EEG devices worn on their heads. All signals recorded in the cloud environment were stored in two different ways: as raw and processed signals.

2. **Measurements of Signals: Empatica E4 & NeuroSky MindWave EEG**

This dataset was created with the aim of conducting a comprehensive analysis of mental fatigue based on various physiological signals. It is well known that creating a diverse data set involves high costs. Reducing this cost will increase the number of observations. At this point, it is an important step to choose devices that are easy to use, easy to implement and cover the targeted data. On the other hand, providing information about cognitive activities and states is another request from the devices to be used. Depending on all these, two different wearable devices were preferred in the study. A hybrid approach was used to determine how mental fatigue is related to both brain, heart, and skin response signals. The signals measuring the heart and skin responses of the individuals were provided by the Empatica E4 smart wristband. Empatica E4 is a wearable health technology device that can measure many biological parameters. It has been preferred in studies on mental fatigue because it provides multimodal sensor data fusion [14]. It allows the harmful effects of mental fatigue on the human body to be monitored with signals obtained from heart and skin responses [15]. It was also confirmed that there is a positive correlation between the same signals obtained in the laboratory environment and the signals generated by this customer wearable Empatica E4 [16]. The signals obtained from this device are blood volume pressure-BVP, electrodermal activity-EDA, heart rate-HR, body temperature-TEMP, 3-axis accelerometer-ACC signals, as given in Table 2. Empatica E4 signals can be easily stored in the cloud with the mobile software application named E4 Realtime.

**Table 2:** Empatica E4 data description.

Empatica E4 data	Sample rate	Description
BVP	64 Hz	It is the signal that measures the heart rate. Heart rate changes that occur when the level of stress or tension is high can be detected with this signal.
EDA	4 Hz	It is the signal data used to measure the electrical activity of the skin. Resistance on the skin surface can vary depending on perspiration, stress, mood, physical activity, and other factors.
HR	1 Hz	It is the signal that measures how many times the heart beats per minute. This signal data is used to track an individual's level of physical activity and general health.
ACC	32 Hz	It is the signal data used to measure movement and activity level. Thanks to this signal data, the activity level, number of steps, mobility status of the individual can be measured.
TEMP	4 Hz	Signal data used to measure the surface temperature of the skin. This signal data is used to monitor how stressed the individual is, exercise and health status.

Another device used in the study is the NeuroSky Mindwave EEG device. NeuroSky MindWave is a type of brainwave measurement device developed to monitor the electrical activity of the brain. This device has been preferred by researchers both in studies in which decomposed brain waves are detected [17] and in studies in which different mental states [18,19] and activities are tracked based on brain signals. There are 4 basic wave types within EEG brain waves. The signal data obtained from these waves are given in Table 3.

3. **Data preprocessing**

**Table 3:** NeuroSky Mindwave EEG data description.

NeuroSky Mindwave EEG data	Frequency range	Description
Delta	0.5–4 Hz	High frequency brain waves during rest and deep sleep
Theta	4–8 Hz	It is characterized as a brain wave that produces a high frequency during the transition to sleep.
Alpha1	8–10 Hz	It is a brain wave, a subdivision of alpha waves, which manifests itself in moments of cognitive relaxation, yawning and a decrease in stress levels.
Alpha2	10–12 Hz	It is a brain wave associated with cognitive concentration and attention and is another subdivision of alpha.
Beta1	12–15 Hz	It is a derivative of beta waves, which are characterized by increased cognitive effort.
Beta2	15–18 Hz	It is another derivative of beta waves. It is a brain wave that enables cognitive alertness and attention, as well as inferring information about memory
Gamma1	30–40 Hz	It is a derivative of gamma brain waves, which are associated with cognitive activities such as attention, memory, problem solving and learning new information.
Gamma2	40–50 Hz	It is a wave type that generates higher frequency than gamma1 waves during cognitive performance such as attention, memory, problem solving and learning new information.
Attention	–	Indicates the level of attention that occurs when an individual focuses on something
Meditation	–	Numerical expression indicating the depth of meditation

The preprocessed version of the created dataset is also presented to be suitable for analysis studies. In datasets containing such physiological signals, the first thing to be done is to ensure the integration of signals with different sampling rates at a standard rate. For this purpose, all signals were subjected to the necessary upsampling and downsampling processes according to 1 Hz, 32 Hz, and 64 Hz sampling rates. The first dataset created by combining the signals adapted according to the frequency sample rate of 1 Hz was named MEFAR_DOWN, the second dataset created by combining the signals adapted according to the frequency sample rate of 32 Hz was called MEFAR_MID, the third dataset created by combining the signals adapted according to the frequency sample rate of 64 Hz was named MEFAR_UP and a preprocessed MEFAR folder was created.

Other preprocessing operations are as follows: linear interpolation technique was applied to complete the missing data. Min-max scaling was used to transform the data. Although we tried to create a balanced dataset by collecting data in the morning and evening, oversampling was applied to the minority class data since the collected signals did not show a balanced distribution. Another important preprocessing step of the study is the labeling of the data. At this point, the 4-point Likert scores of the Chalder Fatigue Scale administered to the participants before the observation were used as ground truth. According to the results of this Likert, a participant with a score of 12 and above is considered both physically and mentally fatigued. This score is applied cut-off value in our study. Each observation was labeled by taking these scores into consideration during the dataset merging phase.

4. **Statistics of investigation**

**Fig. 3:**
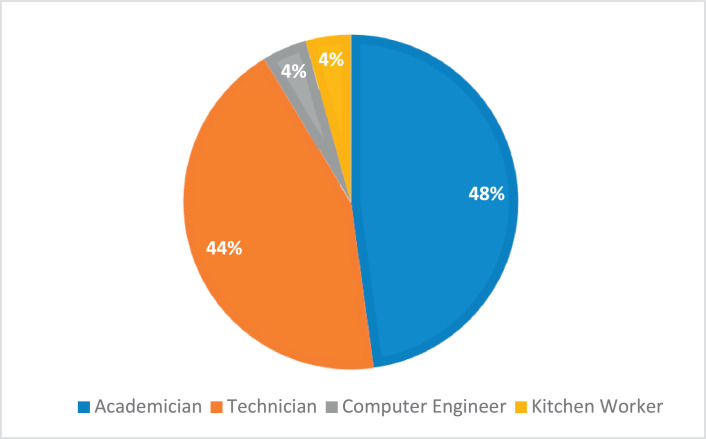
Distribution of subjects' occupation.

Physiological signal data of 23 participants (10 males and 13 females) from 4 different occupational groups were collected to analyze mental fatigue. The primary and only condition for the selection of individuals was to have an active working life. As shown in Fig. 3, 48 % of the participants were from the academician group. The average age of the participants is 29 years and their educational status ranges from primary school graduates to PhD graduates. All the participants are full-time employees and work an average of 8.8 h a day. 39 % of this group of participants, 30 % of whom are married, have regular sleep. Other statistical information is as follows: 26 % smoke, 8 % have chronic diseases, 17 % take regular medication, and 39 % exercise regularly. In the measurements, the average sleep duration of the participants on the last night was 6.5 h.

It was ensured that the duration of each measurement was at least 33 min. In fact, half-hour measurements were targeted and used. However, considering the calibration times of the devices, it was ensured that this period was at least 33. In this direction, data transfer was provided from the devices with a minimum of 33 and a maximum of 36, and 45,540 min of signal measurement was successfully performed.

The answers given by the participants according to the Chalder Fatigue Scale questionnaire were calculated according to the 4-point Likert scale by adhering to the scoring principles of the questionnaire. As a result of these calculations, while 30.43 % of the participants were mentally fatigued in the morning measurements, this rate was 73.91 % in the evening measurements. According to another calculation result, only 10 % of men had mental fatigue in the morning, while 38 % of women had mental fatigue. In the evening, 60 % of men and 84 % of women showed signs of mental fatigue.

## Limitations

While our study represents a significant advancement in the field of multimodal deep learning for evaluating mental fatigue, it is subject to various constraints. The limited sample size of 23 participants limits our capacity to thoroughly investigate individual variations, such as occupation status, that may impact reactions to mental fatigue. Furthermore, the lack of diversity in our participant group limits the applicability of our findings to broader populations. The data we used were obtained by specialized sensors and devices, and discrepancies in these technologies may affect the suitability of our models in various recording environments. The use of the MEFAR dataset for training and testing may restrict the applicability of our findings to different situations, as this dataset may not encompass the complete range of physiological and psychological reactions to mental exhaustion. We strongly recommend further investigation with larger study groups to further explore these elements and maybe uncover more subtle connections. Although there are some limits, our research offers useful insights and sets the stage for future endeavors in this subject.

## Data Availability

MEFAR (Original data) (Mendeley Data)
